# Supplementary material for: Assessing the cost-effectiveness of economic strengthening and parenting support for preventing violence against adolescents in Mpumalanga Province, South Africa: An economic modelling study using non-randomised data
Source: PLOS Glob Public Health. 2023 Aug 17;3(8):e0001666. doi: 10.1371/journal.pgph.0001666 (PMC10434898; doi:10.1371/journal.pgph.0001666)
Supplement: S1 Fig — Cost-effectiveness plane scatter plots of costs per adolescent over effectiveness (in DALYs averted) for four scenarios with the SA GDP per capita as the willingness-to-pay threshold at: A) Routine service costing and population-average prevalence of violence, B) Routine service costing and high prevalence of violence, C) Trial-based costing and population-average prevalence of violence, and D) Trial-based costing and population-high prevalence of violence. (DOCX) [file pgph.0001666.s002.docx]

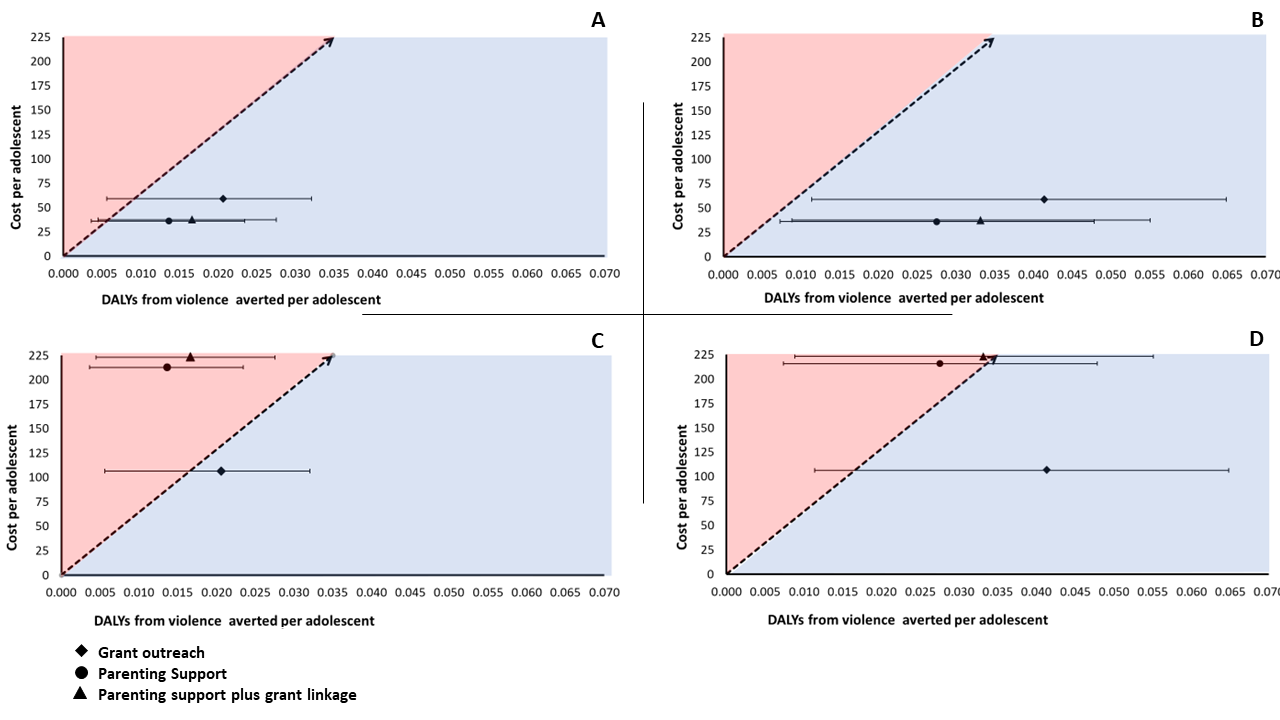


**S1 Fig. Cost-effectiveness plane scatter plots of costs per adolescent over effectiveness (in DALYs averted) for four scenarios with the SA GDP per capita as the willingness-to-pay threshold at: A) Routine service costing and population-average prevalence of violence, B) Routine service costing and high prevalence of violence, C) Trial-based costing and population-average prevalence of violence, and D) Trial-based costing and population-high prevalence of violence. Abbreviations: DALYs, disability-adjusted life years.**
