# Supplementary material for: Assessing the cost-effectiveness of economic strengthening and parenting support for preventing violence against adolescents in Mpumalanga Province, South Africa: An economic modelling study using non-randomised data
Source: PLOS Glob Public Health. 2023 Aug 17;3(8):e0001666. doi: 10.1371/journal.pgph.0001666 (PMC10434898; doi:10.1371/journal.pgph.0001666)
Supplement: S2 Fig — The SA-specific threshold per capita is used as the willingness-to-pay threshold at: A) Routine service costing and population-average prevalence of violence, B) Routine service costing and high prevalence of violence, C) Trial-based costing and population-average prevalence of violence, and D) Trial-based costing and population-high prevalence of violence. (DOCX) [file pgph.0001666.s003.docx]

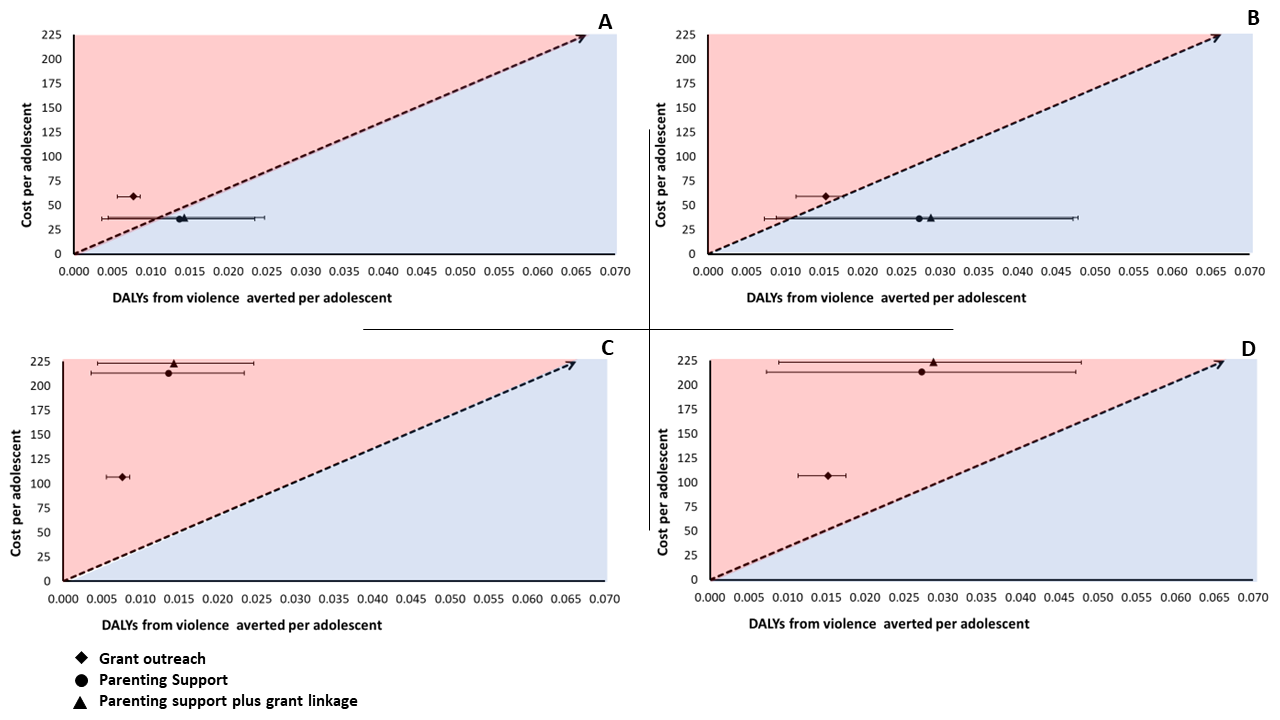


**S2 Fig. Cost-effectiveness plane scatter plots of costs per adolescent over effectiveness (in DALYs averted) for four scenarios using estimates for the relationship between household income and food security drawn from secondary analysis of the South Africa’s 2018 GHS. The SA-specific threshold is used as the willingness-to-pay threshold at: A) Routine service costing and population-average prevalence of violence, B) Routine service costing and high prevalence of violence, C) Trial-based costing and population-average prevalence of violence, and D) Trial-based costing and population-high prevalence of violence. Abbreviations: DALYs, disability-adjusted life years; GHS, General Household Survey.**
