# Supplementary material for: Assessing the cost-effectiveness of economic strengthening and parenting support for preventing violence against adolescents in Mpumalanga Province, South Africa: An economic modelling study using non-randomised data
Source: PLOS Glob Public Health. 2023 Aug 17;3(8):e0001666. doi: 10.1371/journal.pgph.0001666 (PMC10434898; doi:10.1371/journal.pgph.0001666)
Supplement: S1 Table — (DOCX) [file pgph.0001666.s004.docx]

**S1 Table. Description of hypothesised interventions, their duration, and staffing structure.**

|  | **Description** | **Duration** | **Staff structure** |
| --- | --- | --- | --- |
| **Grant outreach** | A one-off community outreach initiative to link all households that are eligible to receive South Africa’s CSG but do not currently receive it.  During the one-off intervention a cadre of paraprofessional social workers would be trained. Once trained, they liaise with community leaders and networks to identify and screen households that may be eligible but not receiving assistance from the CSG and facilitate their access to this initiative.  From expert consultation, we assumed that paraprofessionals would require three household visits to identify an eligible household not currently receiving the CSG, and take two extra days to link an identified household to the Child Support Grant with a success rate of 70%. | 17 months, which corresponds to the estimated time needed to identify all households eligible for the CSG but not currently receiving it.  This was calculated based on the expected number of  household excluded from the CSG, and that hired paraprofessional social workers would be able to reach in a month. | Auxiliary social workers  Coordinators  District co-ordinators  Provincial representative |
| **Parenting support** | Repeating rounds of a 14-session community-based parenting support programme. A cadre of facilitators would be trained. Once trained, pairs of facilitators would support groups of 15 families through the 14-session programme. There would be refresher trainings for facilitators every 3 years. Based on existing scales of parenting support implementation in Thailand and Philippines, we estimated that each year the intervention would be able to reach 2.5% of families living below the upper poverty line in Mpumalanga. | 10 years. Each year there would be three rounds of the 14-session community-based parenting support programme. | Facilitators  Coaches  Assistant coordinators  District coordinators  Provincial representative |
| **Parenting support plus grant linkage** | Repeating rounds of a 14-session community-based parenting support programme, with an additional 15^th^ session focused on linking families that are eligible for the CSG but not currently receiving it to the social services that can help them access it. A cadre of facilitators would be trained. Once trained, pairs of facilitators would support groups of 15 families through the 14-session programme. There would be refresher trainings for facilitators every 3 years. Based on existing scales of parenting support implementation in Thailand and Philippines, we estimated that each year the intervention would be able to reach 2.5% of families living below the upper poverty line in Mpumalanga. | 10 years. Each year there would be three rounds of the 14-session community-based parenting support programme. | Facilitators  Coaches  Assistant coordinators  District coordinators  Provincial representative |

Abbreviations: CSG, Child Support Grant.
