# Supplementary material for: Assessing the cost-effectiveness of economic strengthening and parenting support for preventing violence against adolescents in Mpumalanga Province, South Africa: An economic modelling study using non-randomised data
Source: PLOS Glob Public Health. 2023 Aug 17;3(8):e0001666. doi: 10.1371/journal.pgph.0001666 (PMC10434898; doi:10.1371/journal.pgph.0001666)
Supplement: S2 Table — (DOCX) [file pgph.0001666.s005.docx]

**S2 Table. Summary of data sources used to estimate the effectiveness and cost of grant outreach, parenting support, and parenting support plus grant linkage.**

|  | **Data source** |
| --- | --- |
| **Grant outreach** | |
| Cost | We consulted experts to define an intervention structure necessary to achieve the objectives of this intervention scenario.  Based on expert insight and careful consideration, we defined a reasonable number of household visits that an outreach worker can make in a day, as well as the maximum number of outreach workers that can be employed simultaneously. In our model, we assumed that each outreach worker can conduct three household visits per day and 1000 paraprofessional social workers would be employed in the province.  Furthermore, we incorporated an identification rate into the model, assuming that it takes an average of three visits to identify one household excluded from the Child Support Grant (CSG) program. Once an excluded household is identified, an additional two visits by paraprofessional social workers are required to help these households navigate the administrative processes involved in CSG enrolment.  The unit costs necessary to implement such an intervention scenario were informed by equivalent unit costs obtained from the parenting for lifelong health programme implementation costs (Alampay et al., 2018, & Redfern et al., 2019), which were shared during personal communication with Jamie Lachmann. |
| Effect on food security | The success rate of grant outreach (70%), corresponding to the proportion of eligible households that would be successfully linked to the CSG, was informed by expert consultation and a study by Thurman et al., 2015 that examined the effect of home visiting programme on grant linkage for orphans and vulnerable children in South Africa.  We obtained estimates of the effect of cash transfers on household food security by meta-analysis of effect estimates extracted from two randomised evaluations of cash transfer programmes in Zambia, and Zimbabwe. Further details of this meta-analysis are included in S4 Appendix.  For consistency, we cross-validated these estimates with non-randomized evaluations of the South African CSG on food security, and our own secondary analysis of the relationship between household income and food security using the 2018 South African General Household Survey. Further details of this secondary analysis are included in S5 Appendix. |
| Effect of enhanced food security on emotional, physical, and sexual abuse | These effects were extracted from Cluver and Rudgard et al. 2020 non-randomised evaluation of INSPIRE-aligned protective factors for reducing adolescent violence victimisation. |
| **Parenting support** | |
| Cost | Based on the World Health Organization’s Parenting for Lifelong Health programme, resource needs were identified by following the Parenting for Lifelong Health programme delivery structure of 14 programme sessions, with 15 parent-teen dyads per group.  Unit costs were based on data from the PLH Teen trial obtained from experts (Jamie Lachmann) and included facilitator training and coaching, printed manuals and handbooks, group sessions, home visits, and SMS reminders. Quantities of units required were based on implementation data on parenting programmes in Thailand and Tanzania (provided by experts during consultation).  Capital costs for programme delivery were annualised over their useful lifetime and indirect costs (office space, maintenance, and technical repairs) were included as part of a fixed overhead percentage, assumed in the model to be 10% of the direct costs of the total programme expenditure. |
| Effect on caregiver monitoring score | We obtained estimates for the effect of parenting support on caregiver supervision from the original randomised evaluation of the Parenting for Lifelong Health Teen programme in South Africa. Because there was no evidence that the intervention improved adolescent-reported positive caregiving we only considered the intervention acting via caregiver supervision. |
| Effect of enhanced caregiver supervision on emotional and physical abuse | These effects were extracted from Cluver and Rudgard et al., 2020 non-randomised evaluation of INSPIRE-aligned protective factors for adolescent violence. |
| **Parenting support plus grant linkage** | |
| Cost | Cost of the parenting support component of this intervention were identical to those of used in parenting support only. In addition, we also costed an extra session per wave to the budget of the parenting support intervention. This added cost represents the additional in-session activities, including staff salary, which will be required to identify and assist eligible households to access social assistance. |
| Effect on caregiver monitoring score | We obtained estimates for the effect of parenting support on caregiver supervision from the original randomised evaluation of the Parenting for Lifelong Health Teen programme in South Africa. Because there was no evidence that the intervention improved adolescent-reported positive caregiving we only considered the intervention acting via caregiver supervision. |
| Effect on food security | We obtained estimates of the effect of cash transfers on household food security by meta-analysis of effect estimates extracted from two randomised evaluations of cash transfer programmes in Zambia, and Zimbabwe. Further details of this secondary analysis are included in S4 Appendix.  For consistency, we cross-validated these estimates with non-randomized evaluations of the South African Child Support Grant on food security, and our own secondary analysis of the relationship between household income and food security using the 2018 South African General Household Survey. Further details of this secondary analysis are included in S5 Appendix. |
| Effect of enhanced caregiver supervision and food security on emotional, physical, and sexual abuse | These effects were extracted from Cluver and Rudgard et al. 2020 non-randomised evaluation of INSPIRE-aligned protective factors for adolescent violence.  We modelled food security and caregiver monitoring as combining additively to reduce adolescent violence victimisation, since there was no evidence in the original analysis that they may combine multiplicatively with respect to violence against adolescents. |
