# Supplementary material for: Assessing the cost-effectiveness of economic strengthening and parenting support for preventing violence against adolescents in Mpumalanga Province, South Africa: An economic modelling study using non-randomised data
Source: PLOS Glob Public Health. 2023 Aug 17;3(8):e0001666. doi: 10.1371/journal.pgph.0001666 (PMC10434898; doi:10.1371/journal.pgph.0001666)
Supplement: S3 Table — (DOCX) [file pgph.0001666.s006.docx]

**S3 Table. Estimated DALY per case of physical, emotional, and sexual abuse in South Africa.**

|  | Emotional abuse | Physical abuse | Sexual abuse | Source |
| --- | --- | --- | --- | --- |
| Prevalence of violence among adolescents | 21% | 33% | 4% | Cluver & Rudgard et al. 2020^1^ |
| Total DALYs attributable to adolescent violence | 636,434 | 1,172,331 | 375,097 | Fang et al. 2017^2^ |
| DALY per case of adolescent violence | 0.27 | 0.24 | 0.28 |  |

^1^Cluver LD, Rudgard WE, Toska E, Zhou S, Campeau L, et al. (2020) Violence prevention accelerators for children and adolescents in South Africa: A path analysis using two pooled cohorts. PLOS Medicine. 2020; 17(11): e1003383. doi: <https://doi.org/10.1371/journal.pmed.1003383>

^2^Fang X, Zheng X, Fry DA, Ganz G, Casey T, Hsiao C, Ward CL. The Economic Burden of Violence against Children in South Africa. Int J Environ Res Public Health. 2017;14(11):1431. doi: 10.3390/ijerph14111431

Abbreviations: DALY, Disability-adjusted life years.
