# Supplementary material for: Assessing the cost-effectiveness of economic strengthening and parenting support for preventing violence against adolescents in Mpumalanga Province, South Africa: An economic modelling study using non-randomised data
Source: PLOS Glob Public Health. 2023 Aug 17;3(8):e0001666. doi: 10.1371/journal.pgph.0001666 (PMC10434898; doi:10.1371/journal.pgph.0001666)
Supplement: S4 Table — (DOCX) [file pgph.0001666.s007.docx]

**S4 Table. Detailed breakdown of the cost of grant outreach, parenting support, and parenting support plus grant linkage** **in United States dollar.**

|  | **Parenting Support** | | | **Parenting support plus**  **grant linkage** | |
| --- | --- | --- | --- | --- | --- |
|  | **Routine service costing** | **Trial-based costing** | **Routine service costing** | | **Trial-based costing** |
| **Training costs** | |  |  | |  |
| Pre-programme facilitator training | 14,100 | 36,600 | 14,100 | | 36,600 |
| Refresher course | 4,610 | 15,800 | 4,610 | | 15,800 |
| **Subtotal** | **18,700** | **52,400** | **18,600** | | **52,400** |
| **Programme delivery costs** | |  |  | |  |
| Materials | 19,300 | 173,000 | 19,300 | | 173,000 |
| Pre-programme home visits | 471,000 | 1,150,000 | 471,000 | | 1,150,000 |
| Group sessions | 1,200,000 | 7,390,000 | 1,270,000 | | 7,870,000 |
| During-programme home visits | - | 3,330,000 | - | | 3,300,000 |
| During-programme facilitator coaching | 572,000 | 1,520,000 | 572,000 | | 1,520,000 |
| **Subtotal** | **2,260,000** | **13,500,000** | **2,340,000** | | **14,000,000** |
| **Capital costs** | |  |  | |  |
| Office space and equipment | 226,000 | 1,350,000 | 233,000 | | 1,400,000 |
| **Subtotal** | **226,000** | **1,350,000** | **233,000** | | 1,400,000 |
| **GRAND TOTAL** | **2,500,000** | **15,000,000** | **2,590,000** | | **15,400,000** |
|  | |  |  | | |
|  | **Grant outreach** | | |  |  |
|  | **Routine service costing** | **Trial-based costing** |  |  |  |
| Training of local trainers | 4,100 | 6,100 |  |  |  |
| Training Auxiliary Social Workers | 36,300 | 58,700 |  |  |  |
| Household visits | 3,320,000 | 6,020,000 |  |  |  |
| **GRAND TOTAL** | **3,360,000** | **6,080,000** |  |  |  |
