# Supplementary material for: Assessing the cost-effectiveness of economic strengthening and parenting support for preventing violence against adolescents in Mpumalanga Province, South Africa: An economic modelling study using non-randomised data
Source: PLOS Glob Public Health. 2023 Aug 17;3(8):e0001666. doi: 10.1371/journal.pgph.0001666 (PMC10434898; doi:10.1371/journal.pgph.0001666)
Supplement: S1 Text — (DOCX) [file pgph.0001666.s008.docx]

**S1 Text. Synthesis of the effect of cash grants on household food insecurity in Sub-Saharan Africa.**

We reviewed the literature for studies evaluating the effects of cash transfers on food security using search terms considering these two topics and countries in sub-Saharan Africa. We extracted study findings into an excel spreadsheet. We selected the study outcome that linked most closely to binary measure of food security considered in the non-randomized study of violence prevention accelerators by Cluver and Rudgard et al. 2020, which was the Household Food Insecurity Access Scale (HFIAS), Table A.

**Table A. Summary of the effects of cash transfers on household food security, measured using the household food insecurity access scale (HFIAS).**

| **Author, year** | **Country** | **Programme** | **Monthly grant, USD** | **Sample size** | **Statistical estimator** | **Effect estimate (SE)** | **SMD**  **(SE)** | **HFIAS range** |
| --- | --- | --- | --- | --- | --- | --- | --- | --- |
| Handa et al., 2022^1^ | Zambia | Child Grant | 12 | 6776 | Dif-in-dif at 36 months | 2.43 (0.60) | 0.53 (0.13) | 24 |
| Handa et al., 2022^1^ | Zambia | Multiple Categories Targeting Grant | 12 | 8733 | Dif-in-dif at 36 months | 2.75 (0.51) | 0.54 (0.10) | 24 |
| Bhalla et al., 2018^2^ | Zimbabwe | Harmonised Social Cash Transfer | 10-25 | 1970 | Dif-in-dif at 12 months | 1.19 (0.56) | 0.12 (0.05) | 27 |

^1^Handa, S., Otchere, F., & Sirma, P.; on behalf of the Evaluation Study Team (2022). More evidence on the impact of government social protection in sub-Saharan Africa: Ghana, Malawi, and Zimbabwe. *Development Policy Review*, 40, e12576. <https://doi.org/10.1111/dpr.12576>

^2^Bhalla G, Handa S, Angeles G, Seidenfeld D. The Effect of Cash Transfers and Household Vulnerability on Food Security in Zimbabwe. Food Policy. 2018; 74:82-99. doi: 10.1016/j.foodpol.2017.11.007

Abbreviations: USD United States Dollar, SE standard error, SMD, standardized mean difference; HFIAS, household food insecurity access scale.

We converted all effect estimates to standardized mean differences and used a random effects meta-analysis to generate one overall pooled estimate of the effect of cash transfers on food security, Figure A.

**Fig A. Meta-analysis of the effects of cash transfers on food security.**

Abbreviations: REML, restricted maximum-likelihood; CI, confidence interval; CG, child grant; MCTG, multiple categories targeting grant; HSCT, Harmonised social cash grant.

To correspond with the binary measure of food security considered in the non-randomized study of violence prevention accelerators by Cluver and Rudgard et al. 2020, we then used established methods to convert this pooled estimate of the standardized mean difference to an odds ratio, Table B.

**Table B. Conversion of pooled SMD of the effect of cash transfers on food security to pooled odds ratio and relative risk of the same effect.**

| Pooled SMD (95% CI) | Pooled ln OR (95% CI)^1^ | Pooled OR (95% CI)^2^ | Pooled RR (95% CI)^3^ |
| --- | --- | --- | --- |
| 0.38 (0.09; 0.67) | 0.69 (0.17; 1.21) | 1.99 (1.18; 3.35) | 1.22 (1.06; 1.34) |

^1^Derived using the equation log OR = SMD x (π/√3), ^2^Derived by exponentiating pooled ln OR, ^3^Derived using the equation RR = OR / (1-p0 + (p0 x OR)), where p0 = 0.64.

Abbreviations: SMD, standardized mean difference; ln, natural logarithm; OR, odds ratio; CI, confidence interval; RR, relative risk.
