# Supplementary material for: Assessing the cost-effectiveness of economic strengthening and parenting support for preventing violence against adolescents in Mpumalanga Province, South Africa: An economic modelling study using non-randomised data
Source: PLOS Glob Public Health. 2023 Aug 17;3(8):e0001666. doi: 10.1371/journal.pgph.0001666 (PMC10434898; doi:10.1371/journal.pgph.0001666)
Supplement: S2 Text — (DOCX) [file pgph.0001666.s009.docx]

**S2 Text. Analysis of** **the relationship between household income and food security in South Africa using data from the 2018 General Household Survey.**

To inform a sensitivity analysis of the effect of cash transfers on food security in South Africa, we conducted a secondary analysis of the relationship between household income per capita and food security using data from South Africa’s 2018 General Household Survey. Our aim was to evaluate the relative risk of food security comparing households with an average income per capita, and households with an average income per capita plus the value of a Child Support Grant. Here we provide details of the methodology used for this analysis.

*South African household survey data.* The data source for the analysis was the 2018 South African General Household Survey (GHS). This was chosen as it had a detailed set of household income and food security data. The household dataset was merged with the individual dataset. Observations were restricted to those corresponding with heads of households.

*Measures.*

Food insecurity. Since 2009, the GHS questionnaire has also included a set of four questions based on the Household Food Insecurity Access Scale (HFIAS) to determine households’ access to food. These questions aim to measure households’ food access by asking households about modifications they made in their diet or eating patterns during the previous month because of limited sources available where they can obtain food. They include:

1. Did your household run out of money to buy food during the past 12 months?
2. Did you cut the size of meals during the past 12 months because there was not enough food in the house?
3. Did you skip any meals during the past 12 months because there was not enough food in the house?
4. Did you eat a smaller portion/variety of food during the past year than you would have liked to, because there was not enough food in the house?

Each of these questions is then followed-up with a question asking if the household’s modification to their diet or eating pattern happened “five or more days in the past 30 days”.

We measured food security as households responding ‘Yes’ to any of the above four modifications to household diet or eating patterns five or more days in the past 30 days.

Household income per capita. We measured household income per capita as total household income from salaries/wages/commission, grants, income from a business, remittances, other income (e.g. rental income, interest), pensions, and sales of farm products, divided by the household size.

Covariates. We considered six covariates in our analysis: Household head age, household head sex, household head highest educational qualification, household size, settlement type, and province of residence.

*Data analysis.* Data analysis was conducted in two stages. Both accounted for survey weights and the stratified sampling design of the GHS, and were conducted in Stata/MP 17.0. First, we estimated the association between household income per capita (independent variable) and food security (dependent variable) using multivariable logistic regression adjusting for our six covariates. The analysis was restricted to households living below the 2018 upper poverty line estimated by Statistics South Africa (R 1183) (Stats SA, 2015, Poverty trends in South Africa: an examination of absolute poverty between 2006 and 2015). We assessed the need to restrict our regression analysis to only households in Mpumalanga by testing for evidence of an interaction between household income per capita and province. Finding no evidence of such an interaction we continued to include the full sample, controlling for province. Second, using our multivariable logistic regression model, we calculated the relative risk of household food security at (i) average household income per capita, and (ii) average household income per capita plus a value of income equivalent to the expected increase in household income per capita if all eligible children began receiving a child support grant. The value of this expected increase was calculated by multiplying the 2018 value of the CSG (R 420) by the number of children in each household and dividing this value by the household size. Values of household income per capita were based on averages observed among households with children in Mpumalanga, and our prediction was restricted to the same households in Mpumalanga using an ‘if’ expression with Stata’s margins command.

**Table A. Summary of multivariable association between household income per capita and household food security. Estimates are adjusted for six covariates.**

|  | **aOR (95%CIs), p-value** |
| --- | --- |
| **Household income per capita** | 1.001 (1.001; 1.001), <0.001 |
| **Covariates** |  |
| Household head age | 1.009 (1.003; 1.014), 0.001 |
| Household head sex | 1.096 (0.946; 1.268), 0.221 |
| Education |  |
| No schooling/ Grade 1-4 | 1 |
| Grade 5-7 | 1.311 (1.070; 1.607), 0.009 |
| Grade 8-11 | 1.442 (1.181; 1.761), <0.001 |
| Grade 12/NSC/Higher | 2.582 (1.968; 3.386), <0.001 |
| Household size | 1.006 (0.976; 1.035), 0.714 |
| Settlement |  |
| Urban | 1 |
| Traditional | 0.890 (0.705; 1.125), 0.329 |
| Farm | 1.71 (1.006; 2.920), 0.047 |
| Province |  |
| Western Cape | 1 |
| Eastern Cape | 3.004 (2.014; 4.481), <0.001 |
| Northern Cape | 1.871 (1.171; 2.990), 0.009 |
| Free State | 5.577 (3.505; 8.875), <0.001 |
| KwaZulu-Natal | 4.685 (3.189; 6.880), <0.001 |
| Northwest | 2.466 (1.560; 3.897), <0.001 |
| Gauteng | 3.945 (2.734; 5.694), <0.001 |
| Mpumalanga | 1.717 (1.131; 2.606), 0.011 |
| Limpopo | 15.588 (8.577; 28.333), <0.001 |
| **Constant** | 0.331 (0.206; 0.531), <0.001 |
| N=7095 |  |

Abbreviations: aOR, adjusted odds ratio; CI, confidence interval; NSC, National Senior Certificate.

**Table B. Summary of predicted probability of food security and probability difference comparing the two scenarios (i) average household income per capita, and (ii) average household income per capita plus a value of household income equivalent to household receipt of the child support grant.**

|  | Predicted probability of food security (95%CI) | Probability difference (95%CI) | Probability ratio (95%CI) |
| --- | --- | --- | --- |
| Household income per capita = R 528 | 0.701 (0.646; 0.755) |  |  |
| Household income per capita = R 748 | 0.752 (0.703; 0.803) | 0.052 (0.041; 0.063) | 1.07 (1.06; 1.09) |

Abbreviations: ZAR, South African Rand; CI, confidence interval.
