# Supplementary material for: Assessing the cost-effectiveness of economic strengthening and parenting support for preventing violence against adolescents in Mpumalanga Province, South Africa: An economic modelling study using non-randomised data
Source: PLOS Glob Public Health. 2023 Aug 17;3(8):e0001666. doi: 10.1371/journal.pgph.0001666 (PMC10434898; doi:10.1371/journal.pgph.0001666)
Supplement: Attachment — Submitted filename: Response to reviewers.docx [file pgph.0001666.s010.docx]

**Reviewer #1 Comments to Author**

**The model that authors use is suitable for the issue addressed, however the authors did not give full details of the model which makes it difficult to understand how the results were obtained. They mention model parameters in the document, but the parameters are not listed in one table which makes it difficult for the reader.**

*RESPONSE: Thank you for this feedback. We agree that transparent reporting of our study is essential for clearly communicating how we obtained our results. In our revised manuscript, we have added further details on our model that will increases its interpretability. This includes adding probability estimates drawn from the literature to our probability tree in the methods section (Fig 1). We have also added a full list of the model parameters and their sources in a new Table in the methods section (Table 1). To increase the interpretability of our second figure (Fig 2), we have also summarised the estimated number of averted cases of violence, cost per adolescent, provincial cost, and average cost-effectiveness ratio associated with each intervention scenario in a new table in our results section (Table 3). Finally, we have included a more detailed summary of costs for each intervention scenario in S8 appendix. Further detail on each of these changes is provided in response to additional comments below.*

**The title indicates that the study is on cost-effectiveness of economic strengthening and parenting support for preventing violence against adolescents in South Africa. However, the study is based on data from Mpumalanga province one of the nine provinces in South Africa. Though the authors indicate that Mpumalanga province lie between (in terms of its economic characteristics) the other two provinces Eastern and Western Cape, I feel that the population and economic characteristics in the Mpumalanga province is not a representative of the population in South Africa as a country hence results obtained may not be generalizable to South Africa. I suggest editing the title to reflect that this study is for Mpumalanga province in South Africa.**

*RESPONSE: This is an important point, thank you. The title has now been changed to “Assessing the cost-effectiveness of economic strengthening and parenting support for preventing violence against adolescents in Mpumalanga Province, South Africa: an economic modelling study using non-randomised data.”*

**The introduction is quite long (I suggest editing this section). Lines 52-62 can be in the discussion section. Line 50 to 51 – I suggest deleting this sentence in the introduction. Rather have it in the discussion section as a limitation to this study.**

*RESPONSE: Thank you for your suggestion. In the revised manuscript we have been more concise in our introduction, which has helped to shorten it. In this process, we decided to remove lines 50-51 from the manuscript as the point they raised about decision analytic models being limited by the quality of published evidence is already covered in the limitations section of our discussion. We decided not to move lines 52-62 to the discussion since they help to set the stage for our readers on how we have selected the interventions modelled in our study. Specifically, that existing research finds food security, caregiver supervision, and positive caregiving to be associated with lower odds of multiple forms of adolescent violence victimisation, and that they are therefore promising targets for interventions aiming to reduce adolescent violence victimisation. We believe that without this paragraph in the introduction, the link between the intervention scenarios modelled, and the violence outcomes considered in our study would be unclear.*

“A previous study to guide the choice of ‘best-buy’ INSPIRE strategies in South Africa found that across seven INSPIRE-aligned protective factors, food security, caregiver supervision, and positive caregiving were the most promising targets for addressing multiple adolescent violence outcomes simultaneously (5).”

**The use the word intervention (Lines 112, 122 and 132) is confusing. I suggest using scenario instead of intervention. Each scenario will focus on an intervention or a combination of interventions.**

*RESPONSE: Thank you for your suggestion. We have used ‘intervention scenarios’ in place of ‘interventions’ in lines 111, 121, and 132 and throughout the manuscript.*

**Fig 1. Graphical representation of probability tree model for estimating the effectiveness of interventions on adolescent violence outcomes – more information is needed in this figure. The structure of a tree is not evident. No probabilities are shown in the tree diagram. To enhance understanding of the methods section all necessary information must appear in the tree diagram or must be well explained in the main text. As it stands now it is not easy to replicate this study. This section is the backbone of this research hence it is necessary to show all the information. Alternatively share references where detailed information about the probability tree model can be found.**

*RESPONSE: Thank you for this important point. As summarised in an earlier comment, we have increased the transparency of our reporting by adding* *further detail to the graphical representation of our probability tree in the methods section (Fig 1). We now clearly summarise the probability estimates that we used to model the effect of interventions on intermediary protective factors, and the corresponding effect of the modelled change in intermediary protective factors on adolescent violence victimisation. We also include each estimates source in the figure legend. The probability estimates reported in the probability tree correspond directly with the relative risk ratios for the probability of violence outcomes under different intervention scenarios reported in Table 1.*

**It might be necessary to justify the discount rate used in this analysis.**

*RESPONSE: We agree that this is necessary. For our analysis we adopted a discount rate of 4% as this is more consistent with rates of economic growth experienced in upper middle-income countries, including South Africa, as compared to the discount rate of 3% that is more commonly applied in economic evaluations* *(Haacker et al., 2020).*

“Intervention costs were calculated over 10 years applying an annual discount rate of 4%, as recommended for economic evaluations in upper middle-income countries (35).”

**Line 241 – authors mention that the estimates are for 10 years of intervention. However, there is no period stated for results presented before this line. Does this mean that grant outreach intervention was done over 10 years?**

RESPONSE*: Thank you for your comment. The study’s time horizon was informed by the WHO-CHOICE guidelines for cost-effectiveness analysis. In the methods section, we report our use of a 10-year time horizon with respect to costs of modelled intervention scenarios, and we have also clarified that our estimates for the effects of all three of our intervention scenarios include those accrued during and subsequent to the 10 years of activity.*

*Estimates of the cost of intervention scenarios depended on modelled intervention activities during the 10-year time horizon.* *In the revised manuscript, we have clarified that for grant outreach, this included a one-off 17-month community outreach initiative to link all households that are eligible to receive South Africa’s CSG but do not currently receive it. The grant outreach programme would end once all eligible but excluded households had been visited. For parenting support and parenting support plus grant linkage, this included sequential rounds of parenting support programmes plus or minus grant linkage over the 10 years.*

“For each intervention scenario, we estimated the total cost of modelled intervention activities over 10 years. For grant outreach, this included a one-off 17-month community outreach initiative to link all households that are eligible to receive South Africa’s CSG but do not currently receive it. The grant outreach programme would end once all eligible but excluded households had been visited. For parenting support and parenting support plus grant linkage, this included sequential rounds of 14-week parenting support programmes plus or minus grant linkage over the 10 years. We applied an annual discount rate of 4% to costs, as recommended for economic evaluations in upper middle-income countries (35).”

*For grant outreach, while intervention activities were modelled to last for 17 months, the effects of this intervention scenario were modelled to last longer. Informed by evidence that the effects of cash transfers on food security are likely to be sustained as long as a household receives a cash transfer, we modelled that the effects of newly receiving a CSG would only decline by 3% annually (Haushofer & Shapiro, 2018; Handa et al., 2018). Then, informed by the WHO-CHOICE guidelines for cost-effectiveness analyses, we estimated average cost-effectiveness ratios for intervention scenarios based on activity costs over the 10-year time horizons and all effects occurring as a result of intervention activities during and subsequent to the 10-year time horizon.*

*In our revised manuscript, we also include further details around our assumptions for how sustained the effects of intervention scenarios are on adolescent violence victimisation, the proportion of beneficiaries that drop-out of interventions, and the proportion that age-out of adolescence annually. Effects of receiving the CSG through grant outreach or grant linkage were also modelled to end when adolescents turned 18 and became ineligible to receive this service.*

“For each intervention scenario, first, we used our probability tree model to estimate the relative risk of emotional, physical, and sexual abuse comparing the probability of these outcomes in the presence and absence of cash transfers and/ or parenting support, Table 1. We used the 95% confidence intervals around relative risk ratios to estimate upper and lower bounds around our main effects. Second, we estimated the cumulative number of cases averted due to intervention activities, accounting for an annual dropout rate from grant outreach (2%), an attrition rate from parenting support (3%), an annual aging out effect for grant outreach (13%) and parenting support (11%), and an annual decay in the intervention’s effects on food security (3%) and caregiver monitoring (33%). Effects of receiving the CSG were also modelled to cease when adolescents turned 18 and became ineligible to receive this service.”

**Line 248 – authors say that grant outreach intervention would be expected to reduce the probability of sexual abuse by -0.54ppts (-0.86; -0.16). If the word reduce is used, the negative sign must be deleted. Also edit the sentences after line 248 (all instances where the word reduce/decrease is used).**

*RESPONSE: Thank you for pointing these out. The negative signs have been removed in these instances.*

**There are values quoted in the text which do not appear in the main article tables (see lines 293 to 294). I suggest adding all values discussed in the manuscript in tables appearing in the main text.**

RESPONSE: *Thank you for this suggestion. In the revised manuscript we have added a table summarising the average cost-effectiveness ratios and incremental cost-effectiveness ratios for our modelled intervention scenarios (Table 3). All values discussed in the main text are now included in tables.*

**The low intervention scenario is only mentioned in the discussion section. No mention of such scenario is in the methods section. I suggest adding information on all scenarios in the methods section.**

*RESPONSE: Thank you for pointing out this inconsistency. In our methods section we state that we estimate the effects of intervention scenarios using “average, upper, and lower effect sizes”. In our revised manuscript we have updated the discussion to refer to “lower and upper” effect sizes rather than “low and high” effect sizes.*

“For each intervention scenario, we estimated the number of cases of emotional, physical, and sexual abuse averted per year using relative risk ratios derived from our probability tree. We also estimated upper and lower bounds around our main effects using the 95% confidence intervals for relative risk ratios in our probability tree.”

**Minor Essential Revisions:**

1. **Page 3 Affiliations: The 4th affiliation is not linked to any author. Please edit**
2. **Line 25 Abstract: delete “only”**
3. **Line 27 to 28 Abstract: Last sentence is confusing – cost-effectiveness analysis show that combining the two interventions is second to linking households to grants cost-effective. How does adding a grant linkage component to parenting support enhance cost-effectiveness.**
4. **Line 95 Study setting: Add “South Africa” after “Mpumalanga province”**
5. **Line 164 Fig 1: I suggest making the + sign a superscript.**
6. **Line 272 Parenting support: It should be USD36 (as in Table 3) instead of USD37.**
7. **Line273-274 Parenting support: It’s not clear where the following values are coming from “(USD 275,700 and 1,640,000 annually)”.**
8. **Line 280: Value (2 617 000) is different from what is in Table 3.**

*RESPONSE: Many thanks for spotting these minor revisions.*

1) *The 4^th^ affiliation has now been linked to an author, Prof. Lucie Cluver.*
2) *Line 25 “Only” has been deleted*
3) *We have now specified that this enhanced cost-effectiveness of parenting support plus grant linkage is compared to parenting programmes.*

“Adding a grant linkage component to parenting support is likely to be more cost-effective than parenting support alone.”

4) *Line 94: South Africa has been added after Mpumalanga*
5) *Line 164: + has been changed to a subscript in the figure footnote*
6) *Line 275: Cost of parenting programmes per adolescent has been corrected to be consistent with Table 3.*
7) *These estimates have been calculated as the annual cost of implementing parenting programmes.*

“Grant outreach was estimated to cost USD 59 and USD 106 per adolescent; and USD 3,360,000 and USD 6,080,000 at the provincial level, under routine service and trial-based costing, respectively.”

8) *The value representing the cost of integrated parenting support plus grant linkage at routine service delivery in line 282 and Table 3 are now the same.*

**In the CHEERS list:**

1. **Choice of model – this point is not fully addressed in the manuscript. See Method section comments above.**
2. **Study parameters – not all parameters are in Table 1.**

*RESPONSE: Thank you for highlighting this point. To increase the transparency of our reporting we have added several details to our methods section.*

1. *In the revised manuscript, we have clarified that our choice to use a decision tree model was based on it being a* *simple and logical way for modelling the effect of selected interventions on violence outcomes via intermediary protective factors. We have also provided further details about the model including adding probabilities to the probability tree (Fig 1). We have also clarified that that average, upper and lower effect sizes for interventions correspond to the relative risk of violence and associated 95% confidence intervals derived from contrasting the probability of violence under the modelled scenarios.*
2. *We now report a full list of the parameters included in our model in Table 1 with their sources. These parameters were used to estimate the number of averted cases of violence and average cost-effectiveness ratios for each of our three intervention scenarios.*

**Page 11 Use ‘INSPIRE’ instead of ‘Inspire’**

*RESPONSE: All mentions of INSPIRE have been capitalised.*

**Since the study is based on Mpumalanga province. Is it necessary to list provincial aOR’s for all provinces?**

*RESPONSE: The aim of S7 appendix was to evaluate the relative risk of food security comparing households with an average income per capita, and households with an average income per capita plus the expected value that would be associated with linkage to the Child Support Grant. For this, we conducted a secondary analysis of the 2018 South African General Household Survey data (N=7095 households). First, we evaluated the association between household income per capita and food security using multivariable linear regression. Our regression model included the whole survey sample across South Africa, and was adjusted for six covariates, which we summarise in the supplementary file and included province. The purpose of including the full sample was to increase the precision of our estimates. We agree that if there was evidence of a significant interaction between household income per capita and province, it would have been necessary to restrict our sample to only those households in Mpumalanga or estimate stratum specific effects for this group using the interaction term. However, since we found no evidence that the relationship between household income and food security differed significantly across South African provinces it was valid for us to continue to use the full sample, controlling for province. In the revised supplementary file, we now include details of our test for a significant interaction between household income per capita and province and how this informs our choice to undertake the analysis with the full sample of households.*

**S8: Which one is the lean and ideal scenario? In the main text authors use trial and routine service delivery.**

RESPONSE: *Apologies for this confusion. The use of ‘lean’ and ‘ideal’ relates to a previous version of the manuscript. These terms have both been updated in the revised manuscript − ‘lean’ should have referred to our ‘routine service’ costing strategy, and ‘ideal’ referred to our ‘trial-based’ costing strategy. In our methods we describe that trial-based costing is based on costs drawn directly from the randomised control trial of PLH Teen in South Africa. Routine service costs are based on the expected costs of running each intervention as a routine service. The differences between these two delivery strategies are also described in the methods section.*

*“*For all three interventions, we estimated the cost of interventions using ‘routine service’ and ‘trial-based’ ingredient costing. These two scenarios targeted the same number of families but varied in staff salaries, with the later providing significantly higher salaries, and budgeting for venue hire, printed training materials, and communication with participants via mobile phones. Routine service costing was based on daily salary rates equivalent to South Africa’s average monthly earnings in March 2021, while trial-based costing was based on daily salary rates three times higher than this.”

**S8: The Low effect scenario is not described in the manuscript.**

*RESPONSE:* *Thank you for highlighting this.* *In our methods section we state that we estimate the effect of intervention scenarios using “average, upper, and lower effect sizes”. In our revised manuscript we have updated our results table (now Table 3 in the results section) to refer to “lower and upper” effect sizes rather than “low and high” effect sizes.*

“For each intervention scenario, we estimated the number of cases of emotional, physical, and sexual abuse averted per year using relative risk ratios derived from our probability tree, Table 1. We also estimated upper and lower bounds around our main effects using the 95% confidence intervals for relative risk ratios derived from our probability tree.”

**Reviewer #2 Comments to Author**

**The violence domains the authors explore in this paper could be better and more explicitly defined in the introduction/methods. The introduction mentions adolescents ‘affected by violence,’ but the methods and discussion section more fluidly mixes a bunch of violence outcomes: victimization (e.g., physical and sexual abuse), perpetration (e.g. youth lawbreaking), and an item that is unclear in its mapping to violence (transactional sex). It becomes clearer later on that the authors are only looking at emotional, physical, and sexual abuse, but this seems to be a practical restriction rather than one made based on domains/modes of intervention efficacy. Suggest more clearly defining area of outcome focus throughout. Specifically, the mention of perpetration outcomes and transactional sex in the methods and discussion is confusing.**

*RESPONSE: Thank you for pointing this out, we agree that this is a necessary clarification. We have updated the manuscript to clarify that the aim of our analysis was inform the cost-effectiveness of interventions that prevent adolescent violence *victimization*. Our analysis built on a previous study of protective factors for multiple forms of adolescent violence victimisation by Cluver & Rudgard et al. 2019. According to the aim of our study, we have removed mention of transactional sex, and youth lawbreaking, which – as pointed out by the reviewer − are not clearly forms of violence victimisation. We have also clarified that we were unable to consider community violence victimisation in our analysis, as we could not find any literature on the Disability-Adjusted Life Years (DALYs) attributed to this form of adolescent violence victimisation in South Africa.*

“Third we combined these data in a decision-analytic model to estimate the total DALYs averted, cost, and cost-effectiveness of our selected interventions. This choice enabled us to generate a single estimate of cost-effectiveness for selected interventions, but also limited us to only consider adolescent violence victimisation outcomes with evidence for their attributable DALYs in the published literature. Of the violence outcomes investigated in the non-randomised analysis in South Africa, this included emotional abuse, physical abuse, and sexual abuse, but excluded community violence victimization.”

**Importantly, Interventions 1 and 3 (grant outreach) are only able to assist households that do not already access the Child Support Grant, but this is only a small percentage of the target population. It also seems like the models are parameterized with intervention effects of the cash transfer. So, there is a mixture of interventions and parameterization here that is confusing. I think the CSG is actually the intervention you want to model, in target populations with varying levels of pre-existing uptake. It seems that this is also the cost that is most relevant: the cost of the actual CSG program, not just the cost to link people to the program.**

*RESPONSE: Thank you for this observation. In the design phase of our research, together with experts in adolescent violence prevention, we considered modelling South Africa’s Child Support Grant (CSG) as an intervention scenario. However, we decided that our narrow focus on preventing violence against adolescents would undervalue the cost-effectiveness of this social assistance service, which has much broader aims of helping families meet a collection of basic needs including child education, nutrition, and health.*

*In our analysis, instead of modelling the delivery of the CSG to existing beneficiaries, we decided to evaluate if the investments necessary to link households that are eligible to receive the CSG but, due to exclusion errors, do not access this service, would be outweighed by the cases of adolescent violence victimisation averted amongst households newly receiving cash grants. This decision was informed by previous research published by UNICEF, which estimated that approximately 18% of eligible households may not be receiving the CSG in South Africa. The expert group also agreed that investments in linkage services would be more proportional to the expected number of cases of adolescent violence victimisation that would be averted by intervention activities.*

*In our analysis, we do not to consider the operational costs of delivering the CSG (i.e. the value of grants, overheads, etc.) as it was agreed with experts that these costs are already budgeted for by the South African government in their annual budget projections for the expected number of households that are eligible to receive the grant. As such our analysis assumes that additional investment is needed to link eligible beneficiaries that still do not access the grant they are eligible for, but once they have been linked, the costs associated with them receiving the cash transfer are already assumed by the South Africa Social Security Agency (SASSA). We have clarified that we do not consider the operational costs of delivering the CSG in our revised methods section.*

*“*We did not consider the operational costs of delivering the CSG in our analysis (i.e. the value of grants, overheads) as it was agreed with experts that these costs are already budgeted for by the South African government in its annual projection for the expected number of households that are eligible to receive the grant.”

**Adolescents aged 18 and 19 are not eligible for the Child Support Grant (coverage only goes until age 18), but the target population includes adolescents age 11-19?**

*RESPONSE: Thank you for raising this. In our analysis the population that our findings apply to is adolescents – defined by the UN’s as those ‘between the ages of 10 and 19’. The population that we model as benefiting from the grant outreach intervention scenario is adolescents aged 10-18 based on the Child Support Grant’s eligibility criteria. To do this we include an ageing out effect in our model that accounts for each year a cohort of 17-year olds becoming 18 and therefore ineligible for the Child Support Grant. We have included further details on this in our revised methods section. Additionally, to avoid confusion around the use of ‘Target population’ in our methods section, we have updated this subsection header to ‘Study population’.*

“…we estimated the cumulative number of cases averted due to intervention activities, accounting for an annual dropout rate from grant outreach (2%), an attrition rate from parenting support (3%), an annual aging out effect for grant outreach (13%) and parenting support (11%), and an annual decay in the intervention’s effects on food security (3%) and caregiver monitoring (33%). Effects of receiving the CSG were also modelled to cease when adolescents turned 18 and became ineligible to receive this service.”

“Study population. The study population was households with a monthly household income per capita below the South African upper poverty line of United States Dollar (USD) and an adolescent aged between 10 and 19 years (20-22). In South Africa, the upper poverty line is approximately equal to the means-test threshold used to assess applicant’s eligibility for the national Child Support Grant (CSG).”

**The Child Support Grant is modelled as acting on violence outcomes via food security, which seems rather narrow. The CSG could also plausibly work through, for example, education, mental health, and stress pathways, among others. Similar point is probably true for parenting interventions. Do you allow any of these interventions to work through other pathways besides the limited protective factors specified (food security and positive parenting)? It seems a major underestimate to restrict these holistic interventions to only their effects through narrow intermediate pathways and not their broader set of direct and indirect effects.**

*RESPONSE: This is an important point. In our analysis we only model interventions acting on violence outcomes via improved food security and/or caregiver monitoring as these pathways were identified in a previous analysis to be promising targets for addressing multiple adolescent violence outcomes simultaneously. The three interventions modelled in our analysis are very likely to act on violence victimisation by other pathways beyond food security and caregiver monitoring. We are upfront about this in our abstract. We are also careful to highlight this as a limitation of our study in our discussion, and outline additional evidence-based pathways via interventions may act on adolescent violence victimisation. Finally, we have also added a final sentence in the conclusion stating that further research to account for all of the pathways via which intervention scenarios would act to reduce adolescent victimisation is necessary to increase our confidence in the findings. We are in the process of planning a future study that will more effectively account for the additional pathways via which modelled interventions might act on adolescent wellbeing, including violence victimisation.*

“Several methodological choices also mean that our estimates of cost-effectiveness are likely to be conservative … Also, our focus on the impact of interventions via food security and caregiver supervision alone did not consider the full complement of protective factors via which selected interventions could act on adolescent violence outcomes. There is growing evidence that parenting support is also likely to act on adolescent violence via improving caregiver mental health, caregiver alcohol/drug avoidance, and improved economic welfare (46,52,53). Due to a lack of evidence in the wider literature, we were also unable to consider the wider societal benefits of preventing adolescent violence. Such benefits might include reduced health service use, social service use, and court case time.”

“Further research to account for all of the pathways via which modelled intervention scenarios would act to reduce adolescent violence victimisation is necessary to strengthen our confidence in the findings.”

**On page 7: “For parenting support plus grant linkage, we used our pooled estimate for the effect of cash transfers on food security and our effect estimate for caregiver supervision from the PLH Teen evaluation.” It’s unclear to me whether this pooled estimate incorporates any potential interaction between the two interventions or just adds their effect together?**

*RESPONSE: We agree that this should have been clearer. Since the original analysis did not report an interaction between caregiver monitoring and supervision or food security we simply modelled the combined effect of the two interventions added together. We have provided further detail on this in the methods.*

“We modelled food security and caregiver monitoring as combining additively to reduce adolescent violence victimisation, since there was no evidence in the original analysis that they may combine multiplicatively with respect to this outcome (5).”

**The 10-year implementation timeline strikes me as a bit odd – people in your target population would age in and out of adolescence and out of CSG eligibility over that timeframe.**

*RESPONSE: Thank you for noting this point. We used a 10-year time horizon for our analysis, as recommended in the WHO-CHOICE guidelines for economic evaluations. To account for our target population ageing out of adolescence, in each of our intervention scenarios, we model that each year the population at risk of adolescent violence victimisation declines by a constant rate. Given the dynamic nature of our model, we also account for some additional events that we expect to occur over the 10-year implementation timeline. These include an expected rate of households dropping out of interventions, and an expected annual decay in intervention effectiveness among beneficiaries. We have included further details on these parameters in our revised methods section.*

“…we estimated the cumulative number of cases averted due to intervention activities, accounting for an annual dropout rate from grant outreach (2%), an attrition rate from parenting support (3%), an annual aging out effect for grant outreach (13%) and parenting support (11%), and an annual decay in the intervention’s effects on food security (3%) and caregiver monitoring (33%). Effects of receiving the CSG were also modelled to cease when adolescents turned 18 and became ineligible to receive this service.”

**How is it possible that the grant outreach alone prevents more cases of violence than the grant outreach + parenting support (Table 3)?**

*RESPONSE: Thank you for following up on this. Grant outreach prevents more cases of violence than parenting support plus grant linkage as it has a larger beneficiary population compared to the parenting support plus grant linkage intervention scenario.*

*Grant outreach is directed at all households living below the upper poverty line, and is estimated to result in an additional 57,100 adolescents accessing the CSG. Alternatively, the parenting support plus grant linkage is directed at families that can be reached by parenting support during the study’s time horizon. Based on this, we estimate that this intervention scenario leads to an additional 8200 adolescents accessing the CSG.*

*In the updated manuscript, we detail the different activities and beneficiary populations of the three intervention scenarios in the methods. We also include a summary table at the beginning of the results summarising the different number of adolescents that are linked to the CSG as a result of grant outreach, and parenting support plus grant linkage scenarios. These additional details make our analysis more transparent and clarify why grant outreach prevents a higher number of cases of violence compared to parenting support plus grant linkage.*

*“Scenario 1: Outreach programme to link households that are eligible but not receiving South Africa’s CSG [Grant outreach]. …* in our analysis, we modelled the impact of a community outreach programme implemented over 17-months during which paraprofessional social workers would actively liaise with community leaders and networks to screen for households that are eligible for the CSG in the community. Eligible households not already receiving the CSG would then be supported to access it.”

“*Scenario 3: Integrated parenting support programme plus component to link households that are eligible but not receiving South Africa’s CSG [Parenting support plus grant linkage].* … We modelled the roll-out of a parenting support plus grant linkage programme, structured similarly to the 14-session PLH Teen programme but including one additional session during which facilitators would assess families for their eligibility for inclusion in South Africa’s CSG. Families found to be eligible but not receiving the CSG would be linked to the relevant social services to support them in accessing the grant. This parenting support plus grant linkage intervention would be distinct from the community grant outreach intervention, as it would focus on families that attend the parenting support intervention rather than the broader community.”

“Under the grant outreach intervention scenario, 57,100 adolescents living in 55,500 households would be linked to the CSG. Under the parenting support intervention scenario, 66,900 adolescents living in 65,300 households would attend parenting support. Under the parenting support plus grant linkage scenario, 66,900 adolescents living in 65,300 households would attend parenting support, and 8200 of these would also be linked the to CSG.”

**The conversion of ‘Averted cases of violence’ to DALYs is not presented clearly in the results section.**

*RESPONSE: We agree that this was unclear. In the revised manuscript, we have clarified in the methods section that we used the approach detailed in Redfern et al. 2019 to convert averted cases of violence to DALYs. For this, we first obtained estimates of the DALYs attributable to a case of emotional, physical, or sexual abuse by dividing the total DALYs attributable to each form of violence by the total number of children in South Africa experiencing that same form of violence. Next, we used these estimates to transform the total cases of violence averted into DALYs averted.*

“DALYs attributable to physical, emotional and sexual abuse were calculated from the only known evaluation of the economic consequences of violence against children in South Africa, and estimates of the absolute prevalence of each form of violence in South Africa, S6 Appendix. For full details on this method Redfern et al., 2019.”

**The conclusions in the discussion “Our model indicates that implementing each of our three selected interventions using a routine service delivery model is likely to be cost-effective for reducing emotional, physical, and sexual abuse against adolescents” and the abstract “Findings indicate that investments in community grant outreach, and parenting support interventions are likely to be cost-effective for preventing adolescent violence” don’t seem to align with the findings as reported in the results section. The results seemed to indicate that, by and large, the interventions were not cost-effective (below the WTP threshold for all except under assumptions of high violence)? So, I’m not clear on how these conclusions were arrived at.**

*RESPONSE: Thank you for following up on this. In our analysis, we model each of our three intervention scenarios at a combination of routine service/ trial-based costs and population average/ high prevalence of violence. For each of these combinations, we also generate a lower, middle (best), and upper estimate of cost-effectiveness.*

*Focusing on our middle estimate of cost-effectiveness, which we consider to be our best estimate, we find that all three of our intervention scenarios may be cost-effective if routine service delivery costing is used, whereas, with one exception, none of the intervention scenarios are likely to be cost-effective if trial-based costing is used. Focusing on our upper estimates of cost-effectiveness, we find that these estimates all fall above our willingness to pay threshold even if routine service delivery costing is used and the prevalence of violence among beneficiaries is high.*

*In the revised manuscript, we have updated our discussion and conclusion to more clearly communicate that services may be cost-effective if routine-service costing is used, but not if trial-based costing is used. We also outline that there is uncertainty in this finding since our upper ACER estimates under routine-service costing all cross our willingness to pay threshold. We have also added a final sentence in the conclusion stating that further research to account for all of the pathways via which intervention scenarios would act to reduce adolescent victimisation is necessary to increase our confidence in the findings.*

“Our model indicates that even when modelling intervention effects via food security and/ or caregiver supervision, investments in grant outreach, and parenting support may be cost-effective for reducing adolescent violence victimisation so long as routine service costing is used. Wide confidence intervals around our effect estimates highlight a need for further research before any strong conclusions are drawn. We also find that under routine service costing, adding a grant linkage component to parenting support is likely to be more cost-effective than parenting support alone.”

“**Conclusion.** Investments in grant outreach, and parenting support are most likely to be cost-effective for reducing adolescent violence victimisation if they are provided using routine service costing and they reach adolescents with the highest risk of violence victimisation. We also find that under routine service costing, adding a grant linkage component to parenting support is likely to be more cost-effective than parenting support alone. Further research to account for all of the pathways via which interventions act to reduce adolescent violence victimisation is necessary to strengthen our confidence in the findings.”

**Minor revisions:**

1. **The language around ‘incurring’ DALYs in the intro seems off. My understanding is that they are a measure of years of healthy life lost – suggest updating the language to better align with its definition.**
2. **Small typos and missing words throughout – need copyediting.**

*RESPONSE: Thank you for spotting these minor revisions. They revised wording has been updated and we have corrected typos and missing words throughout the manuscript.*

1. *We have updated the wording.*

“Annually, 1.4 billion children are estimated to be affected by violence worldwide, leading to more than 5.1 million disability-adjusted life years (DALYs).”
